# Using formative research to design context‐specific animal source food and multiple micronutrient powder interventions to improve the consumption of micronutrients by infants and young children in Tanzania, Kenya, Bangladesh and Pakistan

**DOI:** 10.1111/mcn.13084

**Published:** 2020-10-16

**Authors:** Rebecca C. Robert, Rosario M. Bartolini, Hilary M. Creed‐Kanashiro, Allison Verney Sward

**Affiliations:** ^1^ Conway School of Nursing The Catholic University of America Washington D.C. USA; ^2^ Instituto de Investigación Nutricional Lima Peru; ^3^ Global Technical Services Nutrition International Ottawa Canada

**Keywords:** animal source foods, complementary feeding, cultural context, infant feeding, micronutrients, multiple micronutrient powders, qualitative methods, Tanzania, Kenya, Bangladesh, Pakistan

## Abstract

Optimal complementary feeding practices including consumption of multiple micronutrient powders (MNP) are recommended to improve micronutrient intake by infants and young children (IYC) 6–23 months. Formative research was used to design the behaviour change strategy to improve IYC micronutrient intake for the multicountry ENRICH project in rural impoverished areas of Tanzania, Kenya, Bangladesh and Pakistan. Employing a qualitative approach with multiple methods and guided by a social ecological framework, the study was conducted in several phases: data collection in the community, household and health facilities, initial analysis and household trials (HHT). Results found limited use of animal source foods (ASF) for feeding IYC and MNP largely unavailable. Although cost constrained access to ASF, potential more affordable context‐specific ASF options were identified in each setting. Caregivers associated ASF with many positive attributes for IYC, but barriers to feeding them included lack of caregiver time and knowledge of specific preparation techniques, and limited advice from health workers. Feeding practices were identified that used time‐efficient, specific preparations for eggs and other ASF, and demonstrated good initial acceptability and feasibility during HHT. Testing MNP in HHT found good initial feasibility and acceptability and provided an understanding of the facilitators and constraints for preparing, feeding and promoting MNP. In conclusion, formative research led to the design of context‐specific ASF and MNP complementary feeding promotion strategies to improve IYC consumption of micronutrients by identifying the practices, benefits, motivations and alternative actions to overcome the barriers in each setting.

**Key messages**

- Caregivers associated ASF with positive qualities for IYC. Cost, time and preparation barriers to feeding ASF can be overcome by promoting time‐efficient, doable food preparations of context‐specific, less expensive choices such as small fish in Bangladesh and Tanzania, meat and milk in Kenya and chicken or fish in Pakistan.
- Eggs were underutilized across countries yet are a more available and affordable nutritious ASF to feed IYC. Negative perceptions of eggs can be modified with context‐specific preparation methods and promotion of their positive qualities.
- HHT were an important component of the methodological process for understanding the facilitators and constraints for preparing, feeding and promoting ASF and MNP.

## 1 INTRODUCTION

Consumption of nutritious foods and micronutrient supplements at the household level are essential practices for infants and young children (IYC) 6–23 months in low‐ and middle‐income countries (LMIC). For this age group, reaching optimum micronutrient intake requires particular attention, as deficiencies and their associated morbidity occur at the same time that essential micronutrients are needed for brain development and immune functioning—critical for healthy growth and development (Bailey, West, & Black, 2015; Black et al., 2013; Cusick & Georgieff, 2016; Cusick, Georgieff, & Rao, 2018). Globally, iron deficiency represents the most common micronutrient deficiency, and iron deficiency anaemia affects an estimated 41.7% of children <5 years old, with higher regional prevalence in Africa (59.3%) and Southeast Asia (51.4%; WHO, 2020). Other common micronutrient deficiencies in IYC include vitamin A and zinc, and frequently multiple micronutrient deficiencies coexist (Bailey et al., 2015; WHO, 2016). Micronutrient deficiencies reflect a diet of poor quality, often indicated by inadequate dietary diversity at the population level (Working Group on Infant and Young Child Feeding Indicators, 2006).

Among the food groups recommended for children during the complementary feeding period to improve dietary diversity and diet quality are animal source foods (ASF), which include flesh foods (meat, fish, chicken and organ meats), eggs and dairy products (milk, cheese and yogourt) (PAHO/WHO, 2003). ASF are particularly rich in micronutrients (e.g., iron, zinc, vitamin A, vitamin B‐12, among others) with higher bioavailability than plant sources as well as containing high quality protein and essential fatty acids (Bailey et al., 2015; Dror & Allen, 2011; Kwasek, Thorne‐Lyman, & Phillips, 2020; Lutter, Iannotti, & Stewart, 2018; Murphy & Allen, 2003). Flesh foods contain the more bioavailable form of heme iron and additionally enhance nonheme iron absorption from plant sources when consumed together (Hurrell & Egli, 2010). Yet, on a global scale, daily consumption of flesh foods (27.6%) and eggs (16.6%) are particularly low among IYC 6–23 months, and dairy products are consumed by less than half (42.2%) (White, Bégin, Kumapley, Murray, & Krasevec, 2017).

To prevent micronutrient deficiencies and promote optimal micronutrient status in IYC 6–23 months, the World Health Organization (WHO) recommends optimal complementary feeding practices and the use of multiple micronutrient powders (MNP), starting at 6 months (PAHO/WHO, 2003; WHO, 2016). To best promote these practices, intervention programmes designed with a behaviour change strategy are advised (WHO, 2016). Formative research using qualitative methods within local contexts is advocated to provide the needed data and insights to best inform behaviour change strategies for effective complementary feeding interventions (Bentley et al., 2014; Fabrizio, van Liere, & Pelto, 2014). Similarly, formative research is called for to inform context specific programming and communication around MNP (Schauer et al., 2017).

The Enhancing Nutrition Services to Improve Maternal and Child Health (ENRICH) initiative is a multicountry intervention project aiming to improve overall maternal, newborn and child nutrition and health outcomes in rural impoverished areas of Tanzania, Kenya, Bangladesh and Pakistan (World Vision International, 2016). Baseline results from large‐scale surveys in the project sites revealed prevalent undernutrition, poor dietary diversity and lack of MNP consumption but high continued breastfeeding rates at 1 year (ENRICH(a), 2017; ENRICH(b), 2017; ENRICH(c), 2017; ENRICH(d), 2017). Thus, a key goal of the project was to improve micronutrient intake among IYC within each setting. Context‐specific intervention and communication strategies were needed to promote practices aligned with this goal. To design these strategies, formative research was conducted to explore and understand the context‐specific factors and influences around food and complementary feeding within each setting, and the feasibility and acceptability of local possibilities for behaviour change.

Specifically, this study aimed to

- understand the knowledge, perceptions, attitudes and practices around complementary feeding, specifically dietary diversity, ASF and MNP and the barriers and opportunities present within households, health services and communities;
- test the feasibility and acceptability of behaviours and practices to improve consumption of micronutrient rich foods such as ASF and MNP in household trials (HHT); and
- recommend context‐specific practices and communication (for the intervention strategies) to improve micronutrient intakes by IYC.

This paper describes the formative research including HHT related to ASF and MNP for the ENRICH project.

## 2 METHODS

### 2.1 Study design and data collection methods

Following desk reviews of relevant health policy and previous intervention programmes in each country, the formative research commenced using a qualitative approach within a social ecological framework (Stokols, 1996). Research occurred over 1–2 months per country between November 2017 and March 2018 and included several phases and a variety of semistructured methods to engage multiple respondents as described below. Formative research template tools were created in English for each data collection method and adapted for language and context by each country's team (Table 1). Several were based on those in the ProPAN field manual (PAHO/UNICEF, 2013).

**TABLE 1:** Data collection methods and data sources by country

Data collection method	Data source	Country			
		T	K	B	P
Community					
Free‐listing of community foods^a^	IYC caregivers and community members	13	4	10	9
Market survey^b^	Community markets	33	8	4	4
Household					
Qualitative dietary recall for IYC^b^	IYC caregivers	15	12	9	12
In‐depth interviews^a^		23	19	11	12
Food attributes exercise of key foods^b^		15	12	10	12
Home observations of feeding main meal to IYC^b^		14	8	4	0
Household food stock		17	13	0	4
Health services					
Observations of health workers and caregivers during IYC visits	Health facilities	7	7	8	2
Exit interviews from health facility	IYC caregivers	13	21	0	5
Key informant interviews^a^	Heath facility workers; community health workers	15	10	12	3
Household trials					
Household trials^b^	IYC caregivers	18	11	11	3

Abbreviations: T, Tanzania; K, Kenya; B, Bangladesh; P, Pakistan.

^a^Data collection tools provided in Data S1

^b^ Adapted from ProPAN, PAHO/UNICEF (2013).

*Phase 1:* Initially, a free listing exercise was conducted, whereby individual participants (caregivers and various community members) listed all foods eaten in the area by families and by IYC in particular. From these results, a ‘key foods list’ was created with the micronutrient rich foods mentioned, including three groups of ASF: flesh foods, eggs and milk/milk products. The market survey followed, whereby field researchers visited several local markets and shops to observe and talk with vendors about the availability, cost and seasonality of these foods. ‘Food cards’ picturing each of the key food were created for use in Phase 2.

*Phase 2, Part A:* Household research started with a qualitative dietary recall of all food eaten by the child in the previous 24 h (including approximate amounts), as well as the frequency of consuming selected foods in the past week. A semistructured, recorded interview with the caregiver followed and focused on perceptions of healthy growth, aspirations for their child, feeding practices, knowledge and perceptions of MNP and interactions with health workers. In other households, the food attributes exercise was conducted whereby caregivers pile‐sorted the food cards into three groups: foods given to the child already, foods they would consider giving and foods they would not give. The field researcher then explored the specific reasons for placing each food in a group, the age to begin feeding it and its preparation. Home observations focused on meal preparation, interactions between caregivers and IYC, foods consumed, breastfeeding and the home context, using a semistructured guide. Finally, a household food stock tool was applied to identify the foods purchased or home‐produced during the past week.

*Phase 2, Part B:* Health facilities research included semistructured observations to capture the interactions between front‐line health workers and caregivers during IYC visits. These focused on anthropometry, counselling, communication style and delivery of MNP. Field researchers conducted exit interviews with caregivers of IYC leaving the health facility to inquire about what occurred, messages received, MNP receipt and satisfaction with the visit. Key informant recorded interviews with health workers took place at the health facility or in the community and focused on their perceptions of nutrition issues in the area, nutrition counselling, barriers and opportunities to promote optimal IYC feeding and MNP delivery.

*Phase 3:* After completing data collection, an initial data analysis was undertaken to identify potential priority feeding practices and foods to be addressed within each country.

*Phase 4:* Following the methodology of ProPAN, the HHT were implemented to determine caregiver and child acceptability, feasibility and ability to carry out the feeding practices recommended from Phase 3 (PAHO/UNICEF, 2013). Three visits to selected homes in the ENRICH project sites occurred over a short period of time (two in Kenya); a qualitative 24‐hour dietary recall for the child was conducted at the first and last visits. The general structure for HHT visits included an initial visit where the recommended priority practice(s) to test was selected, either assigned by the researcher or negotiated with the caregiver, followed by demonstration and discussion of the practice(s), explaining benefits and using key messages. On the second visit, the caregiver was asked to share her initial experience with the recommended practice(s) and discuss any modifications made and barriers or facilitators encountered. Encouragement to continue the practice(s) was provided. On the final visit the caregiver was interviewed about her experience testing the recommended practice(s).

### 2.2 Setting and sample

The formative research community sites within each country were purposefully selected to maximize sample diversity and represent the important geographical and cultural characteristics of the ENRICH project areas, which included the following:

- Tanzania: Shinyanga and Singida regions
- Kenya: Elgeyo Marakwet County
- Bangladesh: Thakurgaon District
- Pakistan: Sukkur District, Sindh Province

The various respondents and informants were purposefully selected to ensure a diverse sample for each method (e.g., different aged IYC). Sample sizes depended on country and variability within the data (Table 1). Potential participants were identified and introduced via local ENRICH staff or community health workers familiar with the communities.

### 2.3 Data collection

The formative research team members underwent training and pretested the data collection tools. Standard quality assurance procedures were implemented for data collection and analysis including those for obtaining informed consent, maintaining data confidentiality and secure storage of all data. Debriefing sessions were held during the field work.

### 2.4 Analysis

The recorded and transcribed interviews and observation reports were coded following the interview or observation guide topics, and adding emergent codes as needed. A thematic analysis followed using matrices to help organize, reduce, compare and synthesize all of the qualitative data (Miles, Huberman & Saldaña, 2020; PAHO/UNICEF, 2013). Triangulation across methods and informants occurred through discussion of the principal findings, aided by additional matrices to further consolidation. For this paper, we examined data and results across countries, comparing and contrasting the findings.

### 2.5 Ethical considerations

Ethical Review Committee approvals were obtained by the Ethical Review Committee, icddr,b (Bangladesh), the Amref Ethics and Scientific Review Committee (Kenya), the Muhimbili University of Health and Allied Sciences IRB (Tanzania) and the Health Services Academy's Institutional Ethical Review Committee, Government of Pakistan, Ministry of National Health Services, Regulations & Coordination (Pakistan). Each participant gave consent prior to the study activity.

## 3 RESULTS

Several foods were identified for each of the micronutrient rich food groups on the key foods list in each country for subsequent exploration. For the group of ASF, this included a number of local flesh foods, eggs, insects and milk/milk products (Table 2).

**TABLE 2:** Key foods list for ASF by country

	Tanzania	Kenya	Bangladesh	Pakistan
Flesh foods	Meat, chicken, fish, small dried fish (sardines); Shinyanga specific: Termites, liver, cricket; Singida specific: Goat meat, duck, Guinea fowl	Beef, mutton, fish, chicken, insects (termites), liver, intestines	Fish (several varieties), dried fish (small fish, prawn), broiler chicken, duck, mutton, chicken liver	Chicken, fish, mutton, beef
Eggs	Chicken egg	Chicken egg	Chicken egg, duck egg	Chicken egg
Milk/milk products	Milk	Milk	Milk	Milk, lassi, yogourt, rice cooked in milk

### 3.1 ASF availability, cost and purchase

Market surveys demonstrated that a number of animal products were available in each country.

Most were unaffected by season, with a few exceptions (e.g., insects). Cost predominated as the main barrier to obtaining ASF; however, many families still managed to purchase or obtain fish, chicken or meat on occasion. Different varieties of fish lent to price variations with some considered relatively inexpensive, especially in Tanzania and Bangladesh. Eggs were considered a relatively inexpensive ASF option available across all countries. Foods commonly purchased or home‐produced provided insights on access, such as readily available eggs in Kenyan communities where families raised chickens, and the common purchase of small dried fish in almost all Tanzanian households.

### 3.2 ASF attributes

Caregivers generally associated ASF with positive attributes for children. ‘Health’, ‘good for growth’, ‘body building’, ‘strength’, ‘energy, rich in protein’ and ‘vitamins were attributes connected with ASF. In general, chicken, eggs, fish and milk were considered acceptable foods for IYC from about 6–7 months across countries, providing a variety of ASF options for young IYC. Seasonal termites in Kenya were perceived as acceptable for IYC, but the age to feed them varied from 7 to 18 months, whereas feeding crickets in Tanzania was acceptable only after 2 years. Negative perceptions of ASF for IYC included unhygienic milk and fears of choking on meat or fish bones leading some caregivers to feed younger IYC meat soup versus actual meat and to avoid fish.

Exploration of egg attributes demonstrated a more nuanced and context‐specific understanding compared to other ASF (Table 3). Caregivers perceived eggs to carry both positive and negative attributes for IYC, informed by a mix of nutritional properties and cultural influences. For example, in Kenya, concerns included digestive issues for children and uncertainty around excessive protein if too much egg was consumed, or too often; yet ‘rich in protein’ was also considered a positive attribute of eggs. In Tanzania, food insecurity constraints manifested through some caregivers' mention that children fed eggs too often may come to expect them and behave badly when unavailable.

**TABLE 3:** Caregivers' perceived attributes of eggs for IYC by country

	Positive attributes	Negative attributes	Typically fed to IYC from what age	How typically prepared	Common in the household^a^
Tanzania	• Strengthens health • Contains vitamins and protein • Builds body, gives energy, strengthens health • Tasty • Easily available • Good for children	• Expensive in some communities • Children become bad mannered, will steal eggs • Causes stomach‐ache	7 months	Boiled	Sometimes (purchased)
Kenya	• Strengthen the bones of the child • Rich in proteins • Energy giving	• Can affect the digestive system and cause constipation or diarrhoea when too much consumed • Egg is very strong, can cause palpitations	7 months	Boiled and mashed, mixed in porridge, fried with tomato and onion	Yes, purchased or home produced
Bangladesh	• Has all types of nutrition • Has vitamin/calcium • Increases energy • Available at home • Tasty	• Can cause chest congestion	6 months	Boiled and mashed	N/A
Pakistan	• Gives energy • Good for health • Good in winter (hot food)	• Loose stools in summer	7 months	Boiled, fried, or cooked with onion	N/A

Abbreviation: N/A, data not collected.

^a^ Data from the household food stock.

The demands placed on caregivers in all settings—shouldering numerous responsibilities in addition to feeding and caring for the child—emerged as another potential constraint to preparing and feeding ASF (and MNP). They voiced a preference for feeding the child foods prepared for the family instead of cooking separately. Yet they offered specific examples of preparing these family foods with additional attention and care for children such as washing spice off foods, mashing foods to soften them, and separating bones from fish. Although ASF might be available in the family meal, its prioritization for IYC was not ensured. For example, the fish in a typical curry in Pakistan was spread thinly, and the child received little actual fish. Meat prepared for the family in Kenya might not be served to the child if choking was of concern.

### 3.3 ASF in the diet of IYC

Although caregivers linked many positive attributes and beneficial effects of ASF, results demonstrated considerable gaps in their actual practice. Qualitative dietary recall data found a monotonous diet of staple foods with limited diversity common for IYC across countries. Dietary diversity and the practice of feeding ASF was variable and often lacking. Recall of specific food groups from the previous week also found a pattern of low ASF consumption. Kenya was the exception with milk, where caregivers commonly mixed solid food with milk to achieve a soft consistency to feed IYC. Home observations of the main meal fed to IYC found similar results, with ASF infrequently fed.

### 3.4 Responsive feeding

Responsive feeding during mealtime, a feeding style defined by age and developmentally appropriate interactions with IYC by caregivers, was explored because of its importance in ensuring adequate consumption of micronutrient rich foods and MNP (Bentley et al., 2014). During the meals observed, mothers in Bangladesh were involved and helped their children to eat when time permitted; at other times this was delegated to siblings or others. However, encouragement to eat more to meet the child's age appropriate quantity was not observed. In Tanzania and Kenya, younger IYC were more likely to receive encouragement and help in feeding by their mothers whereas older IYC fed themselves and generally received little attention. During interviews in Pakistan, most mothers reported discontinuing feeding once the child stopped eating; offering encouragement to consume more was not mentioned. Qualitative dietary recall data from Pakistan and Bangladesh found that most IYC did not consume age adequate quantities and only about one‐half did in Kenya and Tanzania.

### 3.5 Connecting ASF and MNP to caregiver aspirations for IYC

To explore potential communication strategies around ASF and MNP that resonated with parents, interviews elicited future aspirations for the child and descriptions of healthy children. Parents aspired good education, employment and success in life for their children across countries. In Pakistan, aspirations were more gender based. Parents from all countries readily described both physical and social/emotional dimensions (e.g., ‘gains weight’, ‘energetic’, ‘playful’ and ‘happy’) of healthy children. Providing ‘good, balanced nutrition’ and care was linked to healthy children and achieving aspirations, and ASF were among specific foods mentioned as important to feed. Yet many parents expressed a desire to learn about how to better feed their children and welcomed advice.

### 3.6 Health workers and counselling on ASF and MNP

The role of the health facility workers and community health workers (CHWs) found variable but generally suboptimal counselling practices for complementary feeding. In Bangladesh and Pakistan, children attended health facilities to receive vaccines or for illness. In Tanzania and Kenya, well child visits occurred (i.e., growth, development, vaccines and counselling/education) but in the context of busy, under‐resourced health facilities. If nutrition counselling occurred in any of these settings, health workers commonly used general wording such as ‘feed a balanced diet’ or listed a series of foods the child should eat and rarely inquired about the caregiver's ability to implement the advice. Home visits by CHWs were infrequent across countries and counselling on IYC nutrition, if it occurred, focused on breastfeeding. Few had received training on complementary feeding. Yet many caregivers cited health facility workers and CHWs as trusted sources on feeding IYC and expressed an openness and desire for their advice. Moreover, most health workers identified nutrition counselling as part of their role.

Across countries, MNP were unavailable in most health centres or unreliably stocked and thus, not promoted. Nonetheless, health workers expressed general awareness of MNP and positive perceptions of them, gained from previous training or exposure. In Bangladesh, caregivers had some awareness of MNP as local NGO workers sold them. In Tanzania, a few caregivers reported hearing positive comments about MNP, whereas in Kenya and Pakistan, caregivers remained largely unaware of them.

### 3.7 Initial data analysis

From the analysis of data and triangulation of results in Phases 1 and 2, opportunities and constraints emerged around promoting IYC micronutrient intake and led to the practices recommended for phase 4 (HHT). First, adding context‐specific ASF to IYC diets provided a key opportunity to improve dietary diversity, improve consumption of micronutrients and maximize nutritional impact. Second, the limited availability and awareness around MNP across countries (together with the knowledge that the ENRICH project would supply them) presented an opportunity to promote MNP. Support for promoting ASF came from caregivers who associated positive attributes with feeding ASF to IYC, demonstrated a base of knowledge about a diverse diet and linked this to achievements in life, and their expressed willingness to feed ASF if advised by a health worker. Cost emerged as the principal constraint across countries, but additional factors to consider included time efficient preparations, the use of family foods and the limited practical knowledge for routinely incorporating ASF in the child's diet given the context and low resources available.

Considering these various factors, several context‐specific, doable ASF practices were identified to recommend. In Pakistan, this included separating a piece of fish or chicken in the family curry specifically for the child, or adding egg to potato in order to balance the ‘hot’ property of the egg. In Kenya, shredding meat prepared for the family to accompany the child's ugali was a recommended practice as well as drinking milk. In Tanzania, incorporating the small dried fish easily available in homes into the child's meal and drinking milk were identified. Practices to incorporate eggs in Kenya and Tanzania included tempering how frequently they were given or mixing eggs with other food such as porridge, and including eggs as one of several possible ASF. In Bangladesh, carefully removing the bones from fish and offering eggs or chicken were among the ways to incorporate ASF into diversifying the child's meal. From these recommended practices, several were selected to test for acceptability and feasibility in HHT. For each, context‐specific matrices were developed that included potential barriers and solutions, motivations, methods to demonstrate the practice and messages and benefits to promote them during the HHT, derived from the results of Phases 1 and 2 (PAHO/UNICEF, 2013).

### 3.8 Household trials for ASF

The ASF practices tested included several options in Tanzania (small fish, egg and milk) and Kenya (meat and egg), whereas Pakistan selected eggs to test. In Bangladesh, ASF were part of the practice tested to increase meal diversity for children. In all countries, results showed that the recommended practices were feasible and largely well accepted by mothers and children as shown in Table 4. Mothers remembered the recommended practice, almost all reported implementing the practice at least some of the time, and all intended to continue it. Cost was a limiting factor in some cases, whereby mothers reduced the frequency of giving ASF, and/or opted to give a less expensive ASF option (often eggs).

**TABLE 4:** Household trials with ASF by country

Country and age of IYC	ASF practice tested (sample size)	Selected findings: feasibility and acceptability
Tanzania 7–16 months	Feed eggs, sardines or milk daily (*n* = 13)	• Mothers appreciated having three options. • Most IYC liked ASF except in a couple cases with specific ASF. • Mothers linked ASF to multiple nutrients for children's health.
Kenya 6–23 months	Add an ASF to your child's meal • egg mixed in porridge, or fried/boiled egg • shredded meat (*n* = 11)	• Preparing shredded meat for children was acceptable and allayed concerns about choking. • Eggs viewed a valuable alternative to meat because of lower cost. • ASF linked to child's good health, strength, growth, development. • Easy to practice (eggs).
Bangladesh 7–23 months	Provide the baby with at least 4 types of foods (including ASF such as egg, fish or chicken) (*n* = 6)	• Caregivers found IYC liked and consumed more food when fed a diverse diet; important to help the child consume sufficient quantity at mealtime. • Mothers linked diet diversity to health and growth.
Pakistan 12–23 months	Give egg to the child 3 times a week (mixed with potato) (*n* = 3)	• Child accepted, ‘tasty’, ‘easy to eat’. • Satisfied child ‘did not feel hungry, asked to breastfeed less’. • Egg mixed with potato neutralized the ‘hot’ aspect of egg, becoming a good food for children. • Easy to cook.

### 3.9 Household trials for MNP

Three countries conducted HHT to test MNP with mothers of IYC including Tanzania (*n* = 18), Kenya (*n* = 11) and Bangladesh (*n* = 5). To initially demonstrate MNP use, most field researchers mixed with the sachet with banana as it mashes easily, the colour mixes well and was acceptable by caregivers tasting the preparation and IYC consuming it. Instructions to give MNP followed each country's Ministry of Health recommendations. Results demonstrated that MNP were well accepted and feasible. Mothers correctly recalled the instructions for feeding MNP, almost all implemented the practice, and all but one mother intended to continue the practice. Factors were identified as involved in mothers' experience and process of forming perceptions and opinions about MNP, either as facilitators or constraints regarding this new practice and provided insights for ENRICH's large‐scale implementation plan of MNP (Table 5).

**TABLE 5:** HHT with MNP

Factor	Mother's perceptions: Facilitators and constraints
Ease of use	• MNP were easy and time efficient to add to the child's food (e.g., porridge, ugali or mashed foods such as banana or potato in Kenya and Tanzania) or mixed in a small amount of food (e.g., rice in Bangladesh) to ensure the child consumed it. • Across countries, concern expressed over ensuring the child consumed the portion of food containing MNP.
Father support	• To gain support for this new practice, mothers informed the child's father (Kenya). • Fathers' support facilitated the practice (Tanzania).
Availability/cost	• Limited availability of MNP, a barrier (Kenya, Tanzania). • Cost of MNP, a constraint (Bangladesh).
Child's reaction	• MNP improved children's appetite so they ‘ate well’ (Tanzania, Kenya). • The child's appetite influenced‐‐when good, MNP was easy to feed; when poor, MNP was more difficult to feed (Bangladesh). • Taste of MNP, another barrier (Tanzania, Bangladesh).
Benefits, positive effects	• Immediate benefits were voiced for IYC such as ‘happy’, ‘active’ and ‘played a lot’ (Tanzania, Kenya). These and other longer‐term benefits mentioned by mothers linked MNP to positive effects on health, growth and intelligence.
Social appeal/interest	• MNP generated interest among neighbours who wanted to learn about them (Tanzania). • Some mothers had heard of MNP previously and one had tried them (Bangladesh).
Child's illness	• MNP not given in one case because the child was ill (Bangladesh).
Side effects	• One mother was concerned about diarrhoea, another that MNP would lead to obesity (Tanzania). One mother noted the child's stool turned black (Kenya).
Forget:	• Possibility of forgetting to give MNP because of busy schedules (Kenya, Bangladesh).
Storage:	• MNP sachet loss (rats consumed sachets in one Tanzanian home).

## 4 DISCUSSION

This formative research study identified the context‐specific ASF and MNP feeding practices and communications to design a behaviour change strategy for the ENRICH project promoting increased consumption of micronutrients for IYC in rural impoverished areas of Tanzania, Kenya, Bangladesh and Pakistan. The use of a social ecological framework provided structure to investigate the barriers and opportunities affecting complementary feeding within the community, household and health services. Others have used a similar approach to capture the range of influences and determinants around complementary feeding and ASF in particular (Armar‐Klemesu et al., 2018; Pachón et al., 2007; Pelto, Armar‐Klemesu, Siekmann, & Schofield, 2013; Thorne‐Lyman et al., 2017).

As expected, cost was a prime constraint in all settings. Nonetheless, available, lower cost ASF within the reach of many families (e.g., eggs and fish) were identified, and caregivers fed them when possible, if not daily, during the HHT and expressed their desire to continue this practice. Other studies concur with the constraint of cost but have also identified context‐specific ASF to promote (Armar‐Klemesu et al., 2018; Rasheed et al., 2011; Robert, Creed‐Kanashiro, Villasante, Narro, & Penny, 2017; Sanghvi, Jimerson, Hajeebhoy, Zewale, & Nguyen, 2013; Thorne‐Lyman et al., 2017). Results of the high regard for ASF among caregivers and their acceptability for young IYC from 6 to 7 months across settings importantly supports their promotion from the start of complementary feeding, a time period when IYC diets often lack ASF (Lutter et al., 2018; White et al., 2017). Recognizing the challenge for caregivers to adequately prepare and incorporate these foods into the child's meal lent to recommending specific, doable practices, a facilitator for behaviour change echoed by other studies. For example, in western Kenya, Ahoya, Kavle, Straubinger, and Gathi (2019) describe cooking demonstration interventions that included teaching preparation techniques such as mashing, dicing and shredding foods to encourage improved dietary diversity and ASF consumption. In Peru, food grinders were tested as a low technology solution to prepare meat and fish for IYC and allay caregiver concerns about food texture (Creed‐Kanashiro, Wasser, Bartolini, Goya, & Bentley, 2018).

Feeding eggs to IYC was limited across settings and concurs with other reports of the widespread underutilization of eggs for IYC, particularly in African and Asian countries, despite their general availability and excellent nutritional profile (Iannotti, Lutter, Bunn, & Stewart, 2014; Lutter et al., 2018; White et al., 2017). Iannotti et al. (2014) and Lutter et al. (2018) draw attention to the varying cultural beliefs around eggs that may limit feeding them to IYC, especially younger IYC, but suggest that these barriers are not insurmountable, and our findings agree. By understanding the cultural and context‐specific beliefs and perceptions around eggs in each country, identifying how to incorporate them into IYC diets and effectively communicate this was possible, feasible and acceptable. Similarly, in Ecuador, formative research led to developing a successful social marketing campaign based on local culture to promote eggs (Gallegos‐Riofrío et al., 2018). In India, research found that scrambling eggs overcame the negative cultural perceptions of feeding a ‘hot’ food to IYC (Bentley et al., 2014). In Burkina Faso, the cultural belief that children who consume eggs will become a thief exposed during formative research was targeted by using consistent, focused communication with motivational elements that spoke to local caregivers and promoted feeding eggs (Nordhagen & Klemm, 2018). Recent intervention studies in Ghana and Nepal have increased egg consumption among IYC, showing continued interest in promoting this nutrient rich food. (Broaddus‐Shea et al., 2020; Lutter et al., 2020; Marquis et al., 2018).

Fish were another ASF option identified to promote in three of four countries. Kwasek et al. (2020) report growing interest among LMIC in promoting fish (wild and farmed) because of its rich source of several essential micronutrients as well as high quality protein and essential fatty acids (Tilami & Sampels, 2018). Similar to our study, Thorne‐Lyman et al. (2017) identified community availability, cost and household factors—including cultural beliefs and perceptions about fish for IYC, preparation time and safety as influential determinants for feeding fish to IYC in Bangladesh. Qualitative work in Kenya emphasized the importance of context whereby differing cultural perceptions and acceptance around feeding small fish to IYC were found in two regions (Hotz et al., 2015).

Introducing MNP through HHT provided a useful method to learn about their initial feasibility and acceptability before ENRICH's large‐scale programme implementation. Our findings align with the ‘expanded program impact pathway’ of the various facilitating and limiting factors, designed from a review of MNP literature (Tumilowicz, Schnefke, Neufeld, & Pelto, 2017). The context‐specific foods to mix with MNP, the benefits and motivations for giving them, and the importance of responsive feeding to ensure the portion of food mixed with MNP is consumed were among the informative findings. These findings concur with the recommendations from others to promote MNP within an overall strategy of complementary feeding for best results (Locks et al., 2017; Siekmans, Bégin, Situma, & Kupka, 2017; WHO, 2016; Young et al., 2018).

Health workers faced many challenges in their busy work settings, and realistic interventions and training are necessary to improve their ability to promote micronutrient rich food (e.g., ASF) and MNP, especially as caregivers welcomed and trusted their advice. For example, coordinated communication among all health workers with the use of brief, targeted, consistent messages containing doable actions messages is recommended as well as focused individual counselling on a diverse diet that incorporates ASF and MNP. The literature supports this approach, for example, in Peru, the coordinated use of community health worker visits after health facility visits was key to improving correct feeding of MNP (Creed‐Kanashiro, Bartolini, Abad, & Arevalo, 2016). In Mexico, the design of large‐scale health worker training for IYC nutrition incorporated findings on the demands of health worker routines from formative research (Gonzalez et al., 2019). Sanghvi et al. (2013) described their focus on key behavioural objectives and communication, across various types of media and health workers, identified from formative research, for the successful large‐scale Alive & Thrive programmes in Bangladesh, VietNam and Ethiopia.

The strengths of this study include the extent of comparable data collected in four diverse settings, guided by a social ecological framework, to thoroughly explore multiple perspectives and determinants around IYC micronutrient consumption in each context. Additionally, including HHT as part of the methodological process provided further insights on the selected practices and confidence to promote them.

The limitations of this research are recognized. Although the samples were purposefully selected to represent the diversity in these communities, project areas were extensive, and the small samples may not have captured the full range of perspectives. In Pakistan, access to health facility informants proved challenging and mealtime observations in homes were not accepted, and in Bangladesh, samples were small for caregiver interviews and home observations. HHT protocols varied by country, included small samples, and were of short duration. Although positive outcomes resulted for ASF and MNP consumption during the HHT, ongoing monitoring, support and large‐scale project evaluation is required to determine their sustainability.

## 5 CONCLUSION

ASF and MNP are key sources of micronutrients for IYC, yet consumption remains limited in many LMIC settings. Formative research including HHT led to the design of context‐specific ASF and MNP complementary feeding promotion strategies to improve IYC consumption of micronutrients by identifying the practices, benefits, motivations and alternative actions to overcome the barriers in rural areas of Tanzania, Kenya, Bangladesh and Pakistan. This study contributes to the literature on formative research using qualitative methods to design behaviour change interventions for improving complementary feeding and micronutrient intake in LMIC.

## CONFLICTS OF INTEREST

R. Robert, R. Bartolini and H.Creed‐Kanashiro were hired by the Nutritional International as consultants for this research.

## CONTRIBUTIONS

RCR, RB and HMC‐K designed the research study. ASW oversaw the study implementation. RCR, RB and HMC‐K participated in the analysis and interpretation of the data. RCR and RB wrote the manuscript, and HMC‐K and ASW critically reviewed it.

## Supporting information


**Data S1** Supporting information
