# Supplementary material for: Using formative research to design context‐specific animal source food and multiple micronutrient powder interventions to improve the consumption of micronutrients by infants and young children in Tanzania, Kenya, Bangladesh and Pakistan
Source: Matern Child Nutr. 2020 Oct 16;17(2):e13084. doi: 10.1111/mcn.13084 (PMC7988862; doi:10.1111/mcn.13084)
Supplement: Supplementary file 1 — Data S1 Supporting information [file MCN-17-e13084-s001.pdf]

Appendix A

Tool: Free-Listing of Foods

The free-listing of foods exercise explores foods available and consumed in the area.

In this free-listing of foods exercise you will cover:

- Foods consumed by families including women, infants & young children in the area.

Product: Foods for creating the Key Foods List (nutrient rich foods which will be used for the Market Observations and the Food Attributes Exercises).

Directions:

Conduct the Free-Listing of Foods with key informants. Use the guide below to conduct the exercise.

Sample: At least 4 key informants. To select key informants, you will want those who have knowledge about different foods available/consumed. For example, leaders in the community, community health worker, local nutritionist, knowledgeable women in the community or mothers with young children. Include a variety of key informants in your sample.

Materials needed for the exercise:

- ✓ Tool for the Free-Listing of Foods

INTRODUCTION

Introduce yourself and invite the key informant to participate in the interview by saying, *"We are talking to individuals who are knowledgeable about foods available/eaten in the area."*

OBTAIN CONSENT for the Free Listing: locally determined

Begin with the following questions:

1. I would like you to tell me all the foods eaten in the area. These might be foods on their own or as part of preparations, and foods eaten at different times of the day. We'd like to hear about foods commonly eaten as well as those less commonly eaten. Please include all the foods you can think of. We are interested in all types of foods, for example animal foods like fish and insects, fruits and vegetables, leaves, etc. Probe, any other foods? *(Write down all the foods listed by the key informant in the table below).*

2. Tell me, which times of the year is food most scarce? What do people eat at the times when food is most scarce? And what do people who have the least amount of resources eat at those times?

3. Now, *tell* me about any additional foods given to babies and young children.

Table to write foods as the key informant mentions them.

Foods	Foods

Tool: In-depth Interview with Caregiver of Young Child

Semi-structured interviews use open-ended questions to explore selected topics thoroughly. You will interview caregivers of young children 6-23 months to learn about their perspectives, knowledge, and practices related to feeding of infants and young children (IYC), growth. You will also explore the various roles of others in the household with respect to feeding and cares of IYC. Finally, you will explore caregivers' interactions with the health facility and others in the community (e.g. community health workers) with respect to feeding and care for their IYC.

In this semi-structured interview you will cover caregiver perspectives on:

- Aspirations for their children
- Perceptions of healthy growth
- Infant feeding practices
- Role of various household members with respect to feeding and cares of IYC
- Interactions with health facility workers, community health workers and others (e.g. front-line facility based workers, CHW, traditional midwife, etc.) and the advice received regarding feeding practices and care

Product: Specific feeding practices that need reinforcement or change in the household to promote the practices that favor healthy growth. Opportunities to reinforce or improve the interactions with health facility workers, community health workers and others around feeding and healthy growth. Motivating words/phrases that can be used in counseling about healthy growth promotion. Understanding of caregiving roles in the household

Directions:

Conduct the semi-structure interviews with caregivers (mothers if at all possible) of young children (6-23 months). Tape-record these interviews. Use the interview guide to *guide* the interview.

Sample: 10-15 caregivers (half with IYC 6-11.9 months, half 12-18 months)

Materials needed for the interview:

- ✓ Tool for the In-depth interview with Caregiver of Infants and Young Children
- ✓ Child's growth card
- ✓ Spoke diagrams for household and community members

INTRODUCTION

Conduct the qualitative dietary recall first if it is part of the interview with this mother.

Introduce yourself and invite the principal caregiver to participate in the in-depth interview by saying, *"We are talking to caregivers in this community to learn about how they feed and care for their young children.*

Question	Answer
How old is your child? _______________	If child is 6-18 months, continue with the next question. If the child is out of this age range, thank the caregiver and stop.
What is your child's name? _______________	Refer to the child's name during the interview when you see (name).
What is (name's) birthdate? _______________	Check that age is correct
Mark if the (name) is male or female	Male: ( ) Female: ( )

1. ASPIRATIONS FOR CHILD

First, I would like to talk about the hopes, goals and aspirations you have for (name).

- Tell me, what do you want most for (name)? What are your aspirations or goals for (name's) future?
- What would you need to do, or need to have, in order to achieve these aspirations/goals? Who needs to be involved–only parents, or also the community, the district?

2. PERCEPTIONS of GROWTH and DEVELOPMENT

Now let's talk about (name's) growth and development.

- As a mother you have watched (name) grow from birth. How do you know if (name) is growing and developing well? What things do you look for? Anything else?

3. ROLE AND ADVICE FROM HOUSEHOLD MEMBERS

Now I would like to talk about everyone who lives in the household and what, if anything, their role is in feeding, advising, or caring for (name).

- In the center of this diagram is a circle entitled "Feeding, Advising and Caring for (name)". Now tell me all those who live in the household excluding children (diagram below).
- I am going to write down each of their names and relationship to (name). For example, mother, grandmother, father, etc.

[Complete the diagram, listing all those in the household and their relationship to the child, make sure to include the caregiver you are interviewing].

- Now let's talk about each person you listed; their role in feeding, advising and/or caring for (name). By role I mean what they do, or how they participate in feeding, advising, or caring for the child. It might be that a person has no role, has indirect role (e.g. shops for food), or direct role (e.g. preparing food for the child or feeding the child).

- Of all of those you have listed, who has the most say in feeding and caring for (name)?

- Let's start with (person just named). **[State the relationship to the child for the recording].**

Tell me about his/her role in feeding, advising, or caring for (name). What does he/she do? What advice does he/she provide? Anything else?

- Select another person on the diagram to talk about and repeat the questions **[State the relationship to the child for the recording].**
- Tell me about his/her role in feeding, advising, or caring for (name). What does he/she do? What advice does he/she provide? Anything else?

- Continue with this until all persons listed on the diagram are discussed.

Of the persons you talked about on this diagram that give advice:

- What happens if you don't agree with the advice?
- What happens if you hear different advice from 2 different persons?
- Did you follow the advice you were given? Give me about an example of advice you put into practice.
- Is there advice that you did not follow? Why?

4. ROLE AND ADVICE FROM COMMUNITY MEMBERS

Now I would like to talk about people in the community who provide <u>advice on feeding and care</u> of (name).

- We will fill out a similar diagram, but this time only with influential persons in the community, for example, community health worker, nurse from the health facility, traditional healer, respected leader in the community, etc. (diagram below).

[Complete the diagram, listing community members who are influential in advising about feeding and care of IYC]

- Of those you have mentioned, who is most influential about feeding and care? Why?
- What advice have they provided about feeding and care? What do you think about this advice?
- Did you follow their advice? Why or why not? How often do you interact with this person? **[Make sure the role of each person discussed is clear on the recording-e.g. community health worker].**

- Select another community person on the diagram to talk about and repeat the questions.
- What advice have they provided about feeding and care? What do you think about this advice? Have you followed their advice? Why or why not? How often do you interact with this person?

- Continue until all persons listed on the diagram are discussed.

Of all the persons we have discussed in the <u>household</u> and <u>community</u>, who do you trust most about advice regarding feeding and care of (name). Why? Anyone else?

5. COMPLEMENTARY FEEDING PRACTICES

It is recommended but not necessary that you complete the 24-hour recall with the caregiver first.
Say to the caregiver, *"Now I would like to ask more about feeding (name)."*

ANIMAL SOURCE FOODS

If you completed the recall with the caregiver, copy over the following:

Ate animal product yesterday	YES: ( )	NO: ( )
Ate animal product 5 or more days in past week	YES: ( )	NO: ( )

Let's talk about animal source foods like meat, chicken, fish, insects and eggs.

If you did not complete the qualitative recall with the caregiver, ask the following:

When was the last time you gave (name) animal source foods like meat, chicken, fish, insects or eggs?

Then continue with:

- Tell me, what do you think about giving animal products to young children to eat? Why would you give them? Why wouldn't you give them?
- Has anyone ever advised you to give animal products to (name)? Who?
- What would you think if the health facility worker or community health worker suggested that you give (name) an animal products daily? Why?
- What would need to change to help you give an animal products daily? **OR**
- (if child has met practice) How have you been able to give animal products?
- What would you advise other mothers to do in order to give animal products?

VITAMIN A RICH FOODS

If you completed the recall with the caregiver, copy over the following:

Ate vitamin A rich vegetable or fruit yesterday	YES: ( )	NO: ( )
Ate vitamin A rich vegetable or fruit 5 or more days in past week	YES: ( )	NO: ( )

Let's talk about Vitamin A rich fruits and vegetables like papaya, mango, pumpkin squash and carrots.

If you did not complete the qualitative recall with the caregiver, ask the following:

When was the last time you gave (name) Vitamin A rich fruits and vegetables like papaya, mango, pumpkin squash and carrots?

Then continue with:

- Tell me, what do you think about giving Vitamin A rich foods to young children to eat? Why would you give them? Why wouldn't you give them?
- Has anyone ever advised you to give Vitamin A rich foods to (name)? Who?
- What would you think if the health worker suggested that you give (name) a Vitamin A rich food daily? Why?
- What would need to change to help you give a Vitamin A rich food daily? **OR**
- (if child has met practice) How have you been able to give (name) Vitamin A rich foods?

- What would you advise other mothers to do in order to give Vitamin A rich vegetables and fruits?

THICK CONSISTENCY FOOD or PREPARATIONS AND FREQUENCY

If you completed the recall with the caregiver, copy over the following:

Ate at least 2 thick consistency food preparations (6-8 months) yesterday	YES: ( )	NO: ( )
Ate at least 3 thick consistency food preparations (9--23 months) yesterday	YES: ( )	NO: ( )

Let's talk about the consistency of food preparations and how often they are eaten.

- Tell me, what do you think about giving thick consistency foods to young children to eat? Why would you give them? Why wouldn't you give them?
- Has anyone ever advised you to give thick consistency foods to (name)? Who?
- What would need to change to help you give thick consistency food preparations? **OR**
- (if child has met practice) How are you able to give thick consistency food preparations?

What would you think if the health worker suggested that you give:

- (name, 6-8 months) 2-3 thick consistency food preparations **OR**
- (name, 9-23 months) at least 3 thick consistency food preparations? Why?
- What would you advise other mothers to do in order to give thick consistency food preparations?

QUANTITY of FOOD

If you completed the recall with the caregiver, copy over the following:

Ate approximately ½ cup/small bowl (7 to 8 months) yesterday	YES: ( )	NO: ( )
Ate approximately ¾cup/small bowl (9 to 10 months) yesterday	YES: ( )	NO: ( )
Ate nearly 1cup/small bowl (11 to 12 months) yesterday	YES: ( )	NO: ( )
Ate 1 cup/small bowl (12 to 23 months) yesterday	YES: ( )	NO: ( )

Let's talk about the quantity of food given.

What would you think if the health worker suggested that you give:

- (name) approximately ½ cup/small bowl at 7 to 8 months? **OR**
- (name) approximately ¾ cup/small bowl at 9 to 10 months? **OR**
- (name) nearly 1 cup/small bowl 11 to 12 months? **OR**
- (name) 1 cup/small bowl 12-23 months? Why?
- Why would you give this quantity of food to young children? Why would you not give this quantity of food to young children?
- Has anyone ever advised you to give this quantity of food to (name)? Who?
- What would need to change to help you give this quantity of food? **OR**
- (if child has met practice) How are you able to give this quantity of food?
- And what would you advise other mothers to do in order to give this quantity of food?

CONTINUED BREASTFEEDING

If you completed the recall with the caregiver, copy over the following:

Breastfed child yesterday	YES: ( ) NO: ( )

Let's talk about breastfeeding.

- Tell me, what do you think of breastfeeding for your child? Why would you breastfeed? Why wouldn't you breastfeed?
- What gets in the way of breastfeeding your child? What have you heard gets in the way of breastfeeding young children in this community?
- And what would you advise other mothers to do in order to continue breastfeeding.

DIVERSITY OF FOODS

Let's talk about a few specific groups of foods.

- Tell me, what do you think about giving <u>milk or milk products</u> to young children? Why would you give them? Why wouldn't you give them?
- Has anyone advised you to give <u>milk or milk products</u> to (name)? Who?
- What gets in the way of giving <u>milk or milk products</u> to your child, or to other young children?
- And, what would you advise other mothers to do in order to give milk or milk products.

- Tell me, what do you think about giving <u>beans, seeds and nuts </u>to young children? Why would you give them? Why wouldn't you give them?
- Has anyone advised you to <u>pulses, beans, seeds and nuts</u> to (name)? Who?
- What gets in the way of giving <u>pulses, beans, seeds and nuts</u> to your child, or to other young children?
- And, what would you advise other mothers to do in order to give pulses, beans, seeds or nuts?

SUPPLEMENTS (IRON OR MNP)

If you completed the recall with the caregiver, copy over the following:

Took iron or MNP supplement yesterday	YES: ( ) NO: ( )

Let's talk about iron (or MNP) supplements (depending on what has been available in the area).

- Tell me, what do you think about giving <u>iron</u> (or MNP) to your child, or to other young children? Why would you give iron? Why wouldn't you give iron?
- Has anyone advised you to give iron (or MNP) to (name)? Who?
- What gets in the way of giving iron (or MNP) to your child, or to other young children?
- What would you advise other mothers to do in order to give iron (or MNP).

BIO-FORTIFIED FOODS

If you completed the recall with the caregiver, copy over the following:

Ate a bio-fortified food yesterday	YES: ( ) NO: ( )

Let's talk about the bio-fortified foods ________________

- Tell me, what do you think about giving (the bio-fortified food) to your child, or to other young children? Why would you give them? Why wouldn't you give them?
- Has anyone advised you to give bio-fortified foods to (name)? Who?
- What gets in the way of giving bio-fortified foods to your child, or to other young children?
- What would you advise other mothers to do in order to give bio-fortified foods.

6. INTERACTIONS AROUND FEEDING

Now let's talk more about feeding (name).

- How do you decide when to feed (child's name)?
- How do you decide to stop feeding (child's name) during a meal?
- What happens if (name) stops eating, and you think he/she did not eat enough? What do you do? Does this happen often?
- Who is the person you turn to when you have problems feeding your child? Why? Anyone else?
- What happens when (name) is sick, how do you feed him/her?

7. HEALTH FACILITY VISITS AND USE OF THE GROWTH CARD

Now I'd like to talk about the health facility in your community.

- Have you taken (name) to the health facility?
    - If *YES*, when was last time you went (child's age) and why did you go?
    - If *NO*, why have you not gone?

Think about the last time you took (name) to the health facility for a well-child visit and all the things that happened. How old was (name).

- Do you recall if the health worker weighed (name)? Did the health worker measure (name's) length? Did the health care worker talk about (name's) growth? What did s/he say?
- Did the health worker ask you anything about feeding (name), for example any problems you had or about what foods you had given (name) or if you were breastfeeding (name)? What? Anything else?
- Did the health worker give you any advice about feeding (name)? What advice? Did you find the advice helpful? Why? Did you feel motivated to try the advice at home? Was it possible to carry out the advice you received at home? Why or why not?

Ask to see (name's) **growth card**, if s/he has one**.** Tell me, how does the health worker use (name's) card? Can you explain the different parts of the card? Did the health worker use the card to explain (name's) growth at the last well-child visit?

Note the following from the child's growth card:

	Child's Current Age: _____________
**Type of health facility visit**	**Mark the number of visits**<br>(include age for the well-child visits)
Well-child visit	
Sick-child	
Other	

8. SATISFACTION WITH VISIT TO HEALTH FACILITY

- Were you satisfied with the last well-child visit for (name)? Why or why not?
- What would you suggest to improve visits and your interaction with the health worker?

9. COMMUNITY HEALTH WORKER VISITS

Now I'd like to talk about visits with the community health worker in your community.

- Has the community health worker visited your home?
    - If *YES*, how old was (name) when h/she visited?
    - Tell me about what happened during the visit, and what you talked about.
    - Did the community health worker talk about feeding (name)? What do you remember about what h/she said? Any advice given? Were you able to put this into practice? Why or why not?
    - Do you know when the community health worker is planning to visit next?

    - If NO, the CHW has not visited, ask: What would you think about a community health worker coming to your house to talk to you about feeding practices and the health of your child?
- Have you attended a talk or group session with the community health worker during pregnancy with (name) or since he's been born?
    - If YES, tell me about this session. Were you able to put into practice something that you learned about? Why or why not?

10. DEMOGRAPHICS OF THE CAREGIVER

QUESTION	YES/NO or ANSWER
How old are you?	
Relationship to the child? (e.g. mother, grandmother)	
How many children do you have?	
How long have you lived in this community?	
Do you participate in any feeding or nutrition programs?<br>(List)____	

For section 3 - ADVICE AND ROLE OF HOUSHOLD MEMBERS
(adapted from Audel, J. & Rychtarik, A. Focus on Families and Culture, 2015)

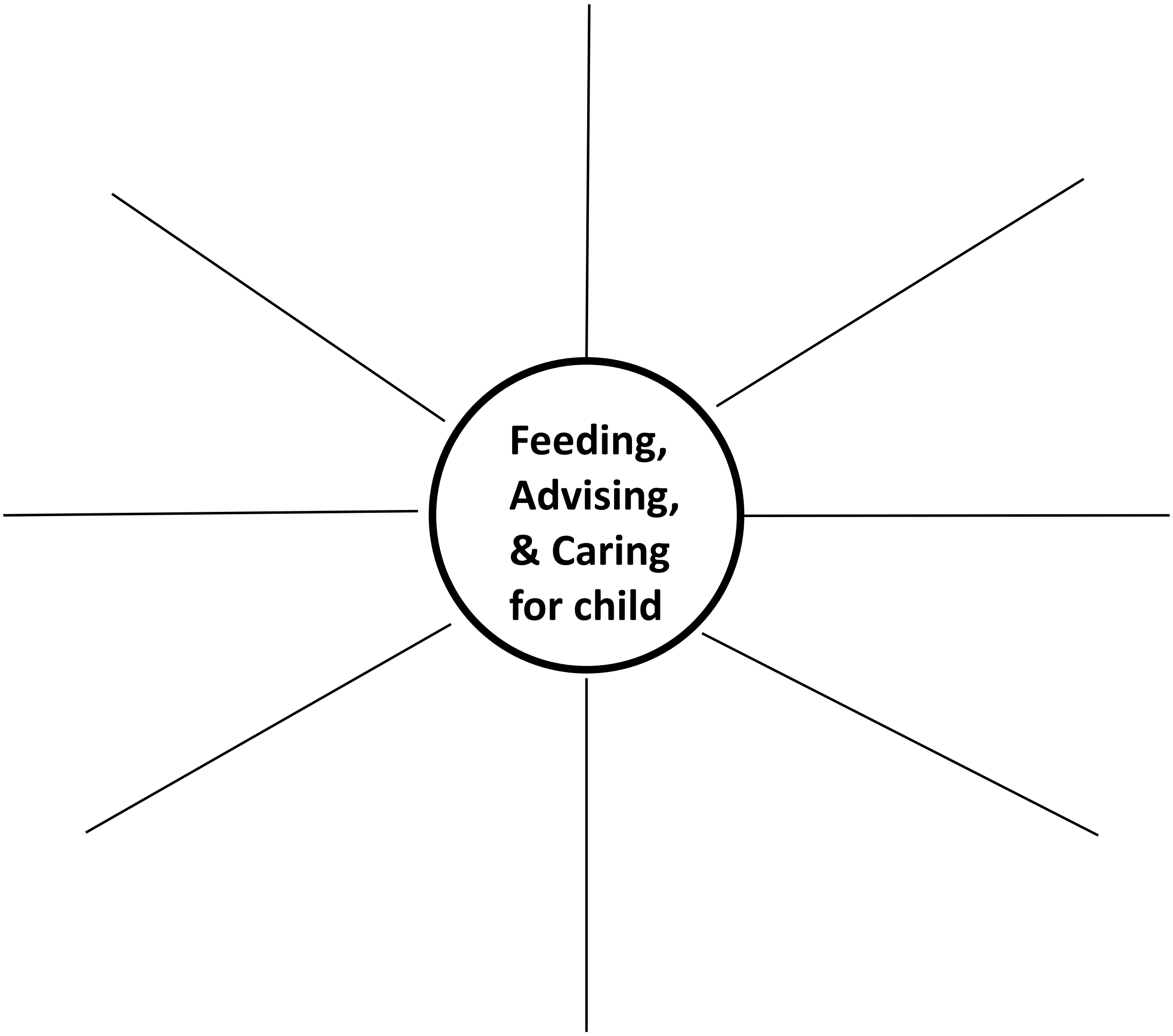

For section 4 - ADVICE AND ROLE OF COMMUNITY MEMBERS
(adapted from Audel, J. & Rychtarik, A. Focus on Families and Culture, 2015)

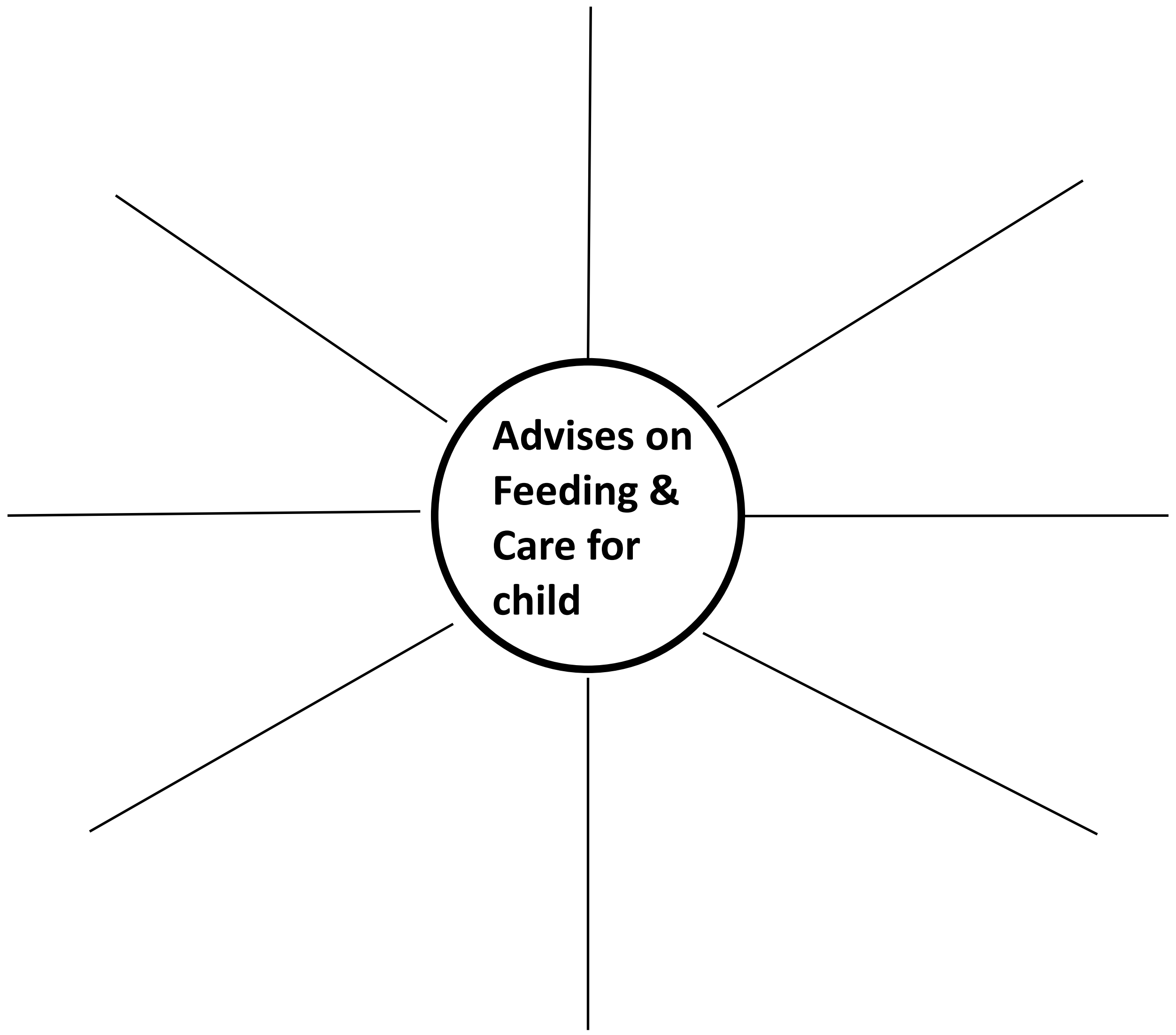

Tool: In-depth Interview with Health Facility Worker of Infants and Young Children

In-depth interviews use open-ended questions to explore selected topics thoroughly. You will interview health workers to learn about their role, perspectives, knowledge, and routines in the health facility.

In this interview you will cover health worker perspectives on:

- Anthropometry measurement routines and explanation of growth
- Common nutritional and feeding problems encountered
- Counseling/delivery of nutrition content and MNP, including communication style
- Barriers and opportunities for promoting ideal feeding practices and growth
- Motivations surrounding their work

Product: Anthropometry and counseling practices to promote, complement or change; health worker knowledge and understanding of breastfeeding, complementary feeding, MNP and growth.

Preparation:
The following will need to be prepared if you complete the optional sections of the interview.

- Take a local growth card and mark the same points on the growth curve as in the Tool (see below, section 2).
- Prepare a dietary recall with local foods using the template from the Tool (see below, section 2).
- Prepare one photo of stunting (2 children born on the same day, but with different heights).

Directions:
Conduct the in-depth interviews with health workers who work with young children. Tape-record these interviews. Use the interview guide to *guide* the interview.

Sample: 1-4 health workers per health facility who have contact with infants and young children

Materials needed for the interview:

- ✓ Tool for the Health Worker Interview
- ✓ Growth card prepared (see above)
- ✓ Dietary recall prepared (see above)
- ✓ Photo of 2 children showing the concept of stunting (optional)

INTRODUCTION

Introduce yourself and invite the health worker to participate in the interview by saying, *"We are talking to health workers to learn about health facility routines and the work with caregivers of young children."*

OBTAIN CONSENT for interview and recording the interview: locally determined

1. WORK HISTORY INFORMATION

Let's start with a few questions about your profession and role in the health facility.

- What is your profession?
- Describe your role in the health facility
- How long have you worked in this health facility?
- Have you received any training on child growth assessment, nutrition counseling or communication (e.g. attended a WHO training course, or MOH specific in-house training, or has not attended training outside of professional curriculum)? If so, what did you receive?

2. (OPTIONAL) EXPLAINING GROWTH AND FEEDING ADVICE

Now I would like to show you the growth card of a child, we will call "Manuel".
Let us pretend that he is a boy in your community who has attended the health facility regularly. Today he is here for his 9 month well-child visit. His weight and length were just recorded (give the prepared growth card to the health worker).

- Would you say Manuel is growing healthy or not? How can you tell? What are the implications for Manuel's health?
- Tell me or show me how you would explain Manuel's growth to his mother today (e.g. use the growth card, other job aid, just talk mother)
- What questions would you like to ask Manuel's mother in relation to his growth? Anything else?

Let's look at what Manuel ate in the last 24 hours (give the prepared dietary recall to the health worker).

Tell me about how Manuel is eating. What are the good things his mother is doing? What are the problems? Why do you think Manuel's mother has these problems? What questions would you would ask Manuel's mother?

As the health worker discusses the dietary recall, you may want to write a few notes in the table below entitled "Guide to check health worker's understanding".

- What advice would you give to Manuel's mother? Anything else?
- If Manuel's mother says he doesn't want to eat, what advice would you offer her? Is this a common complaint from mothers?

Dietary recall of Manuel, 9 months old, who is also breastfed.

**Meal/ Approximate time**	**Register all foods, preparations and drinks consumed. If it is a food preparation, list the ingredients.**	**Consistency of food:** S=Semi-solid/solid L=Liquid/dilute (e.g. soup)
Early morning	One cup of oats with milk and sugar<br>1 tiny bite of bread	L
Mid-morning	A whole banana, mashed	S
Mid-day	Soup with vegetables (vegetable broth, onion, celery, potato,) (1/4 cup broth only). White rice with vegetables from soup (1/2 plate)	L<br><br>S
Afternoon	Drink of tea with sugar	L
Evening meal	Soup with vegetables (vegetable broth, onion, celery, potato) (1/2 cup broth only)	L
Throughout day	Breastfed	
	**Total number of S=semi-solid/solid meals**	**2**

Guide to check health worker's understanding:

**Feeding practice**	**Feeding practice would be considered <u>Good</u> or <u>Inadequate</u>**	**HW identified feeding practice** *(yes/no and comments)*
Manuel was breastfed	Good	
Manuel did not eat an animal source food yesterday	Inadequate. A child should have an animal source food daily	
Manuel had 2 meals with solid/semisolid (S) preparation	Inadequate. A 9 month old child should have 3 "S" meals	
Manuel did not eat a Vitamin A rich food	Inadequate. A child should have a Vitamin A rich food daily	
Manuel was fed an adequate quantity in the morning, but not in the evening	Inadequate. While he ate an adequate quantity for 2 meals, a 9 month old child should have an adequate quantity at 3 meals	

4. DESCRIBING NUTRITIONAL PROBLEMS IN THE COMMUNITY, THE ROLE OF THE HEALTH FACILITY, BARRIERS AND OPPORTUNITIES:

Let’s continue to talk about the children in your community.

- What are the main nutritional or growth problems you see in children? Are many children affected by these?
- What are the effects of the nutritional and growth problems you describe?
- What do you think are the main barriers to children growing healthy in your community? Anything else? How do you and other health workers at your facility deal with these obstacles?
- What helps children in your community to growth healthy?
- Are you aware of any bio-fortified foods in the area? What is your opinion of them? Are they consumed by IYC? Would you recommend that IYC consume them? Why?

Now let’s talk specifically about feeding. What are the main feeding problems you see for:

- children 0 – 5.9 months old
- children 6 – 7.9 months old
- children 8 – 11.9 months old
- children 12 – 23.9 months

(OPTIONAL):

Now I am going to show you a picture of 2 children, born on the same day, but now older.
(use photo of 2 children born on the same day but of different heights).

- What do you notice about these 2 children? What are the reasons behind this difference? Anything else?
- Is stunting a problem in this community, or in the children you see at the health center?

5. HEALTH FACILITY ROUTINES

Now I would like to understand the routines in your health center:

- What is the recommended schedule for well-child visits for infants and young children? Do caregivers bring their children to these visits in your community? Are there certain visits that are better attended than others? Explain. Why else do they come to the health facility?
- Describe the usual routine during a well-child visit (ie. who the caregiver sees or what health workers are involved, the routines for anthropometry, consultation, vaccines, provision of MNP or iron, etc.)
- What is your role with respect to these well-child visits?

About Anthropometry (if not covered above):

- When (for what type of visits) are children weighed?
- When are they measured? Is there a length board in the clinic?
- When is MUAC measured?

- Who in your health facility usually weighs and/or measures children? And, who talks to the mother about growth? Anyone else?
- Does your health facility have goals with respect to child nutrition and growth? Explain them.
- What statistics, if any, are you required to report to MOH/district office related to growth?

6. COUNSELING ABOUT FEEDING, MNP, BARRIERS AND OPPORTUNITIES

- Who in the health facility is responsible for talking with caregivers of young children about feeding?
  - What, if any, differences exist among the role of various health workers for talking with caregivers about feeding?

If the health worker being interviewed counsels caregivers, continue with this section; if not, skip the rest of this section and go to the next section: Describing motivations expectations, and frustrations of health workers.

Now I'd like to ask you about the feeding advice given to caregivers of young children 0-23 months. Just to be clear when I talk about complementary feeding, I'm referring to feeding children 6-23.9 months.

- Tell me, how are caregivers usually counseled about feeding? Walk me though an example.
- In your opinion, what are the most important feeding practices for healthy growth?
  - For infants 0-6 months
  - For infants 6-7 months
  - For infants 8-11 months
  - For infants and young children 12-23 months
- Are MNP or iron supplements provided to IYC in your facility? At what age? Are MNP or iron supplements always stocked (mark which one or both)? (IF PROVIDED, CONTINUE TO INCLUDE MNP/IRON QUESTIONS; IF NOT EXCLUDE THESE QUESTIONS)
  How are caregivers counseled about (MNP/iron)? What concerns do caregivers voice about their IYC taking MNP/iron?
- Are job aids used when counseling caregivers about breastfeeding or complementary feeding? Are growth cards with the caregivers? Can you show me how you use them (job aids/growth cards)?
- What are the biggest obstacles you face in talking to caregivers of young children about breastfeeding? Complementary feeding? Anything else? Have you found a way to overcome these obstacles? Explain. What is easiest to talk about when advising caregivers about feeding?
- What are the biggest obstacles you face in talking to caregivers of young children about taking **MNP/iron**? Have you found a way to overcome these obstacles?
- In general, how difficult or easy is it for caregivers in this community to <u>follow</u> your advice on breastfeeding? Complementary feeding? What specific difficulties do caregivers have in following your advice, and how do you deal with these? What have you found to be most effective way to counsel a mother about feeding? Can you think of a particular young child, and tell me about your experience?

- How difficult or easy is it for caregivers in this community to follow your advice on giving **MNP/iron** to their IYC?
- Among the health workers in your facility, how consistent is the advice about breastfeeding? complementary feeding? That is, do you know what other health workers say or how they counsel caregivers? Do health workers give the same messages or do they give different messages? How consistent are health workers in the manner they counsel caregivers? Explain.
- How consistent is the advice about taking **MNP/iron** among providers?

7. COMMUNICATION WITH COMMUNITY HEALTH WORKERS (CHW)

Now, I'd like to hear about your role with the community health workers in this community.

- Tell me about any community health workers that work with your health facility? What health areas do they cover? What do they do? How do they report to the health center? How do you communicate with CHW? (e.g. in person, phone, texting, other technology, etc.)
- What is your role with respect to the community health workers? (eg. accept referrals, involved in meetings/trainings, receive information about number of IYC with nutritional problems, etc).
- Are there aspects of the CHW role that you would like to improve? What? How do you think you can best work with CHW to promote IYC ideal feeding practices and growth in your community?

8. DESCRIBING MOTIVATIONS, EXPECTATIONS, AND FRUSTRATIONS OF HEALTH WORKERS

Finally, I'd like to hear about the motivations and frustrations in you work.

- Tell me about what is most satisfying or rewarding about your work? Now tell me about the frustrations with work? What suggestions do you have to improve your work?
- Tell me about staff rotation in your health facility? How often does this happen. What effect does this have on routines in the health center? What happens with new staff—how do they learn the routines at this health center?

Anything else you would like to add to or say about our interview today?

Growth Chart of Manuel (example)

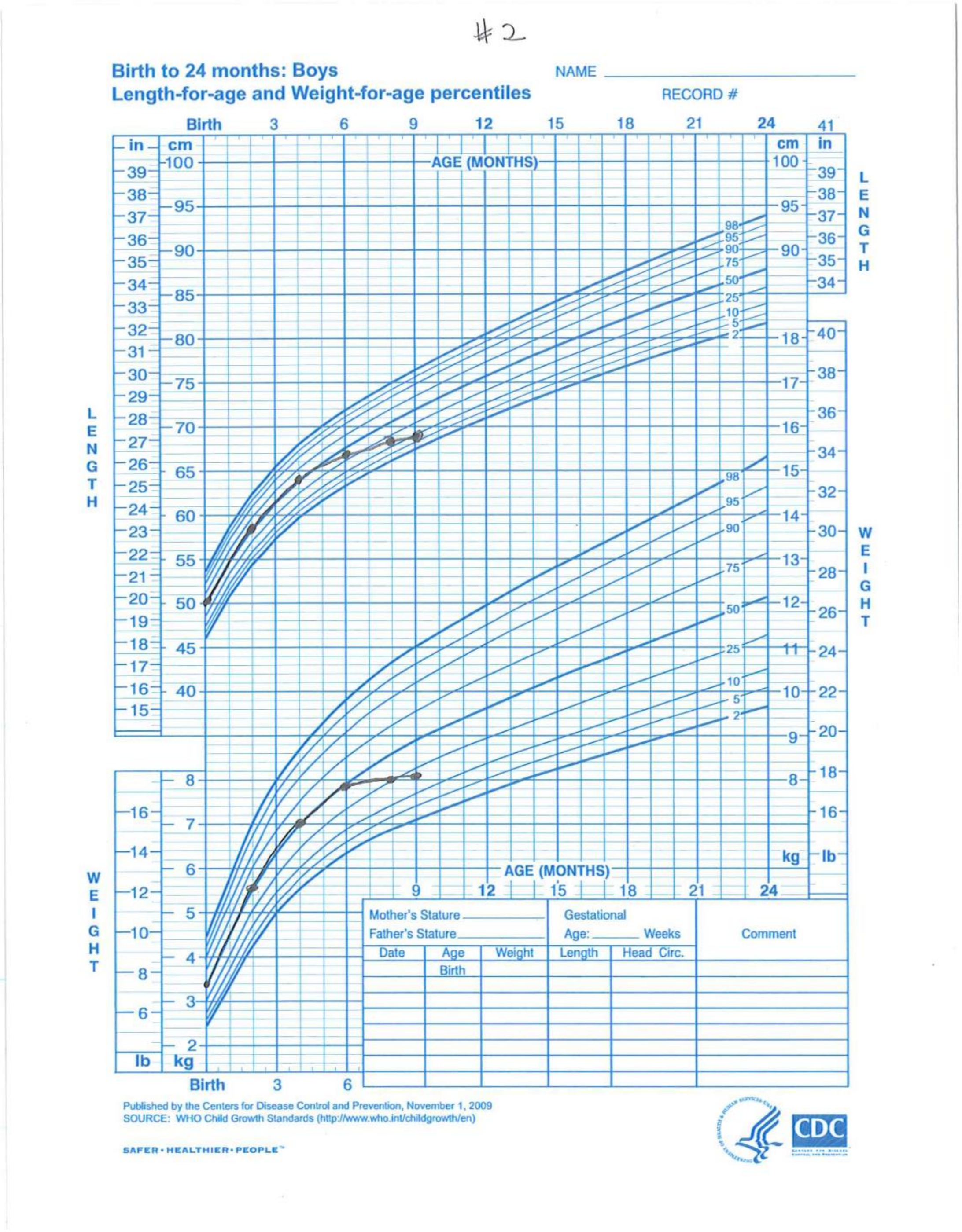

Tool: In-depth Interview with Community Health Workers

In-depth interviews use open-ended questions to explore selected topics thoroughly. You will interview community health workers to learn about their role, perspectives, knowledge, and routines.

In this interview you will cover community health worker perspectives on:

- Common dietary/nutritional problems encountered in pregnancy and lactation
- Common feeding/nutrition problems encountered in IYC
- Role in the community with pregnant women and IYC
- Delivery of nutrition advice for pregnant women and IYC in the community
- Communication and role with respect to health facility
- Motivations surrounding their work

Product: CHW practices to promote, complement or change; community health worker knowledge and understanding of pregnant and lactating women's dietary needs, IYC feeding practices, MNP and IFA, bio-fortified foods, and other recommendations.

Directions:
Conduct the in-depth interviews with community health workers who work with pregnant women, infants and young children. Tape-record these interviews. Use the interview guide to *guide* the interview.

Sample: 2-3 community who have contact with pregnant women, infants and young children 0-23 mo.

Materials needed for the interview:

✓ Tool for the Community Health Worker Interview

NOTE: This interview guide covers both pregnant women and IYC as some CHW may provide care to both groups. The guide is quite long and we recommend several strategies:

i) Conduct the interview in 2 stages, first about IYC and second about pregnant women;
ii) Conduct separate interviews for CHW who work with children, and CHW who work with pregnant women;
iii) Conduct separate interviews with CHW by topic (e.g. first part of the interview including IYC and pregnant women, second part of the interview including IYC and pregnant women).

INTRODUCTION

Introduce yourself and invite the community health worker to participate in the interview by saying, *"We are talking to community health workers to learn about their work with pregnant women, infants and young children in this community."*

OBTAIN CONSENT for interview and recording the interview: locally determined

1. WORK HISTORY INFORMATION OF THE COMMUNITY HEALTH WORKER

Let's start with a few questions about your work as a community health worker (CHW).

- How long have you been a CHW?
- How were you selected for this position?
- Have you received training about child growth assessment, nutrition counseling, antenatal care or communication? Tell me about the topics of any trainings you have recently attended.
- Tell me about why it is necessary to have a CHW in your community?

2. CURRENT ACTIVITIES AS CHW – pregnant women and IYC

Now let's talk specifically about the activities you do as a CHW.

- Tell me what you do as a CHW. Anything else?
- Do you visit homes? Which homes do you visit? What do you do during these visits?
- Do you visit the homes of <u>pregnant women</u>? What do you do during these visits? What, if anything, do you do around nutrition? IFA consumption? Tell me an example of two where you have provided nutritional advice for pregnant women and what you said.
- Do you visit the homes of <u>IYC</u>? What do you do during these visits? What, if anything, do you do around feeding practices? Growth? Have you provided advice on feeding practices as a CHW? What kind of advice have you given? Tell me an example or two where you have provided advice on feeding practices and what you said.

3. INFORMATION MANAGEMENT – pregnant women and IYC

I'd like to learn about how you manage information about your community.

- How many families are in your community? How many children under 5 years of age? Under 2 years? How many pregnant mothers are there?
- How do you know the number of children and pregnant women in your community? Why is it important to know this information? What is it needed for?
- How do you keep track of this information?

****

4. INFANTS AND YOUNG CHILDREN IN YOUR COMMUNITY– HEALTH

Let's talk about the children in your community.

- How is the health of children in your community?
    - What do you consider to be the main health problems of children in your community?
    - What concerns you most?

- Who is involved in children's health in the community?
    - Do the community leaders/authorities participate in the health of IYC? How?

    - Do you ever meet with mothers and/or fathers to learn about the problems of children in your community? How often?
    - Who else in the community does something for the health of IYC? What do they do?

- What cares/activities do parents do for their children to keep them healthy?
    - What do they do in the home? What do they do outside of the home?
    - Whom do they rely on for advice?
        - In what case would parent consult with traditional healers?
        - In what cases do you consult with health facility workers?

5. INFANTS AND YOUNG CHILDREN IN YOUR COMMUNITY – NUTRITION

Now let's talk about nutrition for IYC in your community.

- Tell me, what are the main nutritional problems for children that exist in your community? How many children are affected by these problems, many or a few?
- What are the main feeding problems in your community? Do these problems change by age of the child? Why do you think these problems happen?
- Tell me about breastfeeding practices in your community.
- Tell me about typical feeding practices for children 6-23 months (complementary feeding) in your community.

6. INFANTS AND YOUNG CHILDREN IN YOUR COMMUNITY - HEALTH FACILITY VISITS

Let's talk about attendance at <u>well child visits</u> in the health facility.

- How common is it for mothers in your community to bring their children to the health facility for well child visits?
- What happens at the well child visits?
- Why do some mothers take their children to these visits? What makes it easy for them to attend well child visits?
- Why do some mothers <u>not</u> take their children to these visits? What makes it difficult for them to attend well child visits?
- In your opinion, how important is it for mothers to take their child to the health facility for well child visits? Why?
- Should the community leaders/authorities worry about whether mothers take their children to the health facility for well child visits? Why? If yes, what can they do?

7. INFANTS AND YOUNG CHILDREN - NUTRITIONAL ADVICE BY HEALTH FACILITY AND OTHERS

Now I'd like to ask you about the feeding advice given to caregivers of young children 0-23 months.

- Who in the community provides advice to mothers about breastfeeding, and feeding practices (complementary feeding) to their children. What advice is given?
- What about at the health facility – do health worker provide advice? What do they say?
- What about in a typical home? Who generally provides advice about feeding?

8. INFANTS AND YOUNG CHILDREN - BIO-FORTIFIED FOOD AND SUPPLEMENTS

Now I'd like to talk about bio-fortified foods and supplements for infants and young children (IYC).

- Are bio-fortified foods consumed by IYC? Would you recommend that IYC consume them? Why or why not?

- Tell me about any iron supplements that IYC take. Who takes them and when? Where do they receive the supplements? Why do they take them? What do you think about iron supplements for IYC? What positive comments have you heard about them from families? What negative comments have you heard from families?
- What problems do mothers have in giving iron to their IYC? (eg. supply not available, worried about side effects, etc.)
- Are there case in which consumption of iron has been good? What facilitated this?
- Have you ever had a role in promoting iron consumption for IYC? Tell me about this.

- Are you aware of multi-micronutrient powders (MNP)? What do you know about them? Have you received any training about MNP? What is your opinion of them? Are you aware of any children receiving MNP from the health facility?

  (Per the baseline MNP has not yet been introduced yet in the community, so unlikely that CHW will know about them)

****

14. COORDINATION AND COMMUNICATION WITH HEATLH FACILTIY WORKERS AND OTHERS – pregnant women and IYC

Now, let's talk about coordination and communication with the health facility workers

- How is your relationship with health facility workers (e.g. nurses)?
- How do you coordinate/communicate when them? What about? Where? How often? Do you use any type of technology to communicate (eg. phone call, texting, other).
- How often do they come to the community? With whom do they coordinate to make community visits? What do the health facility workers do when they visit the community? How do you participate with the health staff when they visit the community of homes in the community?
- Do you make any follow-up with mothers? In their homes?
- Has the health personnel coordinated with you or someone else to track and monitor the nutrition and/or health status of pregnant women in your community? How about for IYC?
  - If yes, tell me about how this works. Do you use any technology to help you track or monitor pregnant women or IYC?
  - If no, would you be interested in some type of a system like this? What are your ideas on how this could work?

Tell me, how is your relationship with the community authorities?

- What do they think of your role?

- How do you coordinate with them?

Are there NGOs in the area working on IYC nutrition or health? What about nutrition or health of pregnant women?

- Which NGOs? And what are they doing?
- What is your relationship with these NGOs? Do you coordinate anything for them?

15. MOTIVATIONS AND FRUSTRATIONS OF COMMUNITY HEALTH WORKERS

Finally, I'd like to hear about the motivations and frustrations in you work.

- Tell me about what is most satisfying or rewarding about your work? Now tell me about the frustrations with work? What suggestions do you have to improve your work?

Is there anything else you would like to add to or say about our interview today?

Thank you very much for your collaboration today.
